# Consistency between anticholinergic burden scales in the elderly with fractures

**DOI:** 10.1371/journal.pone.0228532

**Published:** 2020-02-24

**Authors:** Luis Fernando Valladales-Restrepo, Marlene Duran-Lengua, Edgar Eduardo Castro-Osorio, Jorge Enrique Machado-Alba

**Affiliations:** 1 Grupo de Investigación en Farmacoepidemiología y Farmacovigilancia, Universidad Tecnológica de Pereira-Audifarma S.A, Pereira, Risaralda, Colombia; 2 Grupo Biomedicina, Fundación Universitaria Autónoma de las Américas, Pereira, Risaralda, Colombia; 3 Universidad de Cartagena, Cartagena, Bolivar, Colombia; 4 Internal Medicine, Geriatrics, Grupo de Investigación en Farmacoepidemiología y Farmacovigilancia, Hospital Universitario de Caldas, Manizales, Colombia; 5 Grupo de Investigación en Farmacoepidemiología y Farmacovigilancia, Universidad Tecnológica de Pereira-Audifarma S.A, Pereira, Risaralda, Colombia; Annunziata Hospital, ITALY

## Abstract

**Objective:**

Falls and bone fractures are important causes of morbidity and mortality in the elderly. The objective of this study was to identify the degree of consistency between the anticholinergic scales used for patients diagnosed with fractures.

**Methods:**

This was an analytical agreement study conducted in patients diagnosed with vertebral and nonvertebral fractures in Colombia. The quadratic-weighted *kappa* coefficient was used to identify the consistency between the Anticholinergic Drug Scale-ADS, Anticholinergic Cognitive Burden Scale-ACB and Anticholinergic Risk Scale-ARS in assessing the prescriptions of fracture patients during the month prior to the fracture, during their stay as an inpatient and at discharge, according to Landis criteria.

**Results:**

220 patients with fractures were included, with a mean age of 75.3±10.3 years, and 68.2% were women. The ACB scale identified the highest anticholinergic burden (26.8%) in prescriptions made the month before the fracture, and the highest agreement was between ACB and ADS (0.717); during hospitalization and at discharge, the cholinergic antagonists were best identified with ADS (77.7% and 72.1%, respectively), with the best agreement between ACB and ARS (0.613 and 0.568, respectively). The prescription of tramadol was found in 64.1% of hospitalized patients and in 61.4% of patients at the time of discharge.

**Conclusions:**

The scales evaluated show marked discrepancies between them, with highly variable frequencies of anticholinergic drugs identified at the different prescription times, and with low agreement among them, which is why the scales are not interchangeable in patients with bone fractures.

## Introduction

The aging population is a global phenomenon that poses several challenges for medical care. With the expected increase in the number of pathologies and the high use of medications in the geriatric population, it is essential to have an adequate understanding of the quality of prescriptions in this age group [1]. Polypharmacy is associated with the development and worsening of geriatric syndromes, including falls [2] and is involved in the increase in adverse drug reactions (ADR), hospitalizations, complexity of care, morbidity and mortality [3].

Falls and fractures are one of the main causes of morbidity and mortality in the elderly. One in three older adults will have at least one fall each year, and a Swedish study showed that 7% of falls in the elderly result in fractures [4]. Fractures are associated with complications, which in turn prolong hospitalization and increase the risk of institutionalization, morbidity and mortality [5].

The risk of falls increases significantly in relation to the amount of drugs consumed, regardless of age or disability, especially drugs with anticholinergic properties such as benzodiazepines and diuretics [6]. It has been identified that between 9.1% and 50% of the geriatric population uses some drug with anticholinergic properties [7, 8], which are also associated with an increased risk of cognitive impairment, delirium, hallucinations, impulsive behavior, falls, fractures and increased mortality [8, 9].

In Colombia, it was identified that prescribed anticholinergic drugs increased the risk of hip fractures (odds ratio (OR): 1.97, 95% confidence interval (CI):1.19–3.27) [10]. In the United States, it is estimated that problems related to medication cause 106,000 deaths and cost 85 billion dollars annually [11].

Due to the wide use of these drugs, it is recommended to use instruments to quantify the exposure of anticholinergic and sedative drugs, to improve the quality of the prescription by reducing polypharmacy and the risk of adverse reactions associated with their use [12].

Anticholinergic risk scales are tools used to estimate the anticholinergic burden of medications prescribed to a patient. Such scales usually classify drugs in a range of 0 to 3 points according to their anticholinergic potential, from none (0) to strong (3) potential. In all scales, the total anticholinergic burden is determined by the sum of the score of each anticholinergic drug [13–15]. The best validated instruments are the Anticholinergic Drug Scale (ADS), Anticholinergic Cognitive Burden Scale (ACB) and Anticholinergic Risk Scale (ARS) [16].

However, there is considerable variation among the anticholinergic burden scales, especially in terms of antimuscarinic potency and the number of drugs considered in each instrument [16, 17], and there is no current consensus on which scale is optimal [18]. There are few studies that have compared the agreement between anticholinergic burden scales, which have shown in several clinical contexts generally low or intermediate consistencies [19–22]; moreover, it is unknown which scale identifies the highest proportion of anticholinergic drugs in patients with fractures and the variability or agreement between the instruments is unknown, especially since some of the drugs considered in the risk scales are not available in all countries. Therefore, the aim of this study was to identify the degree of agreement between the anticholinergic burden scales in patients diagnosed with fracture in Colombia.

## Materials and methods

This was a prospective, analytical, agreement study comparing three anticholinergic burden scales in patients diagnosed with vertebral and nonvertebral fractures. The anticholinergic burden were analyzed by the following instruments: ADS, ACB and ARS scales one month before the fracture, during hospitalization and at discharge, in the period from January 1 to June 30, 2018. All patients with fracture of any sex who were 60 years or older were selected if they received care in one of the three high complexity hospitals in Colombia. Fractures that were due to infectious etiology, to oncology, secondary to traffic accident and by firearm were excluded.

The information was collected through a survey addressed to each patient after signing an informed consent form and by accessing their medical record. The data recorded included the following variables:

- Sociodemographic: sex, age, city of origin, high complexity hospitals.
- Clinical: type of fracture (vertebral and nonvertebral), number of comorbidities (grouped into 3 categories: 0 pathologies, 1–3 pathologies and ≥4 pathologies).
- Pharmacological:
  - Polypharmacy: prescription of 5–9 drugs. Excessive polypharmacy: 10 or more drugs.
  - Anticholinergic drugs. A search was made of 88 of the 117 drugs included in the ADS scale, 64 of 88 included in the ACB scale, and 43 of 49 in the ARS scale that are marketed in Colombia, according to the National Institute for Food and Drug Surveillance (Instituto Nacional de Vigilancia de Medicamentos y Alimentos, INVIMA) [23]. The total anticholinergic burden was determined by the sum of the risk of each of the prescribed medications. Based on this data, the patients were classified into three groups: 1. Patients with a score of 0 (without anticholinergic activity); 2. Patients with a score 1 and 2 (mild-moderate anticholinergic activity); and 3. Patients with a score ≥3 (high anticholinergic activity).

The protocol was approved by a Bioethics Committee of the Universidad Tecnologica de Pereira, in the category of risk-free research (code: CBE-SYR-162016). The ethical principles established by the Declaration of Helsinki were respected. Personal data of the patients were not considered. Authorization was obtained from each of the reference centers involved in the investigation.

The data were analyzed with the statistical package SPSS Statistics, version 24.0 for Windows (IBM, USA). A descriptive analysis was carried out with frequencies and proportions for the qualitative variables and measures of central tendency and dispersion for the quantitative variables. The X^2^ test was used to compare categorical variables. The dependent variable used in the binary logistic regression models was the use of outpatient drugs with an anticholinergic burden according to the ADS, ACB and ARS scales, and the covariables were those variables that were significantly associated with these drugs in the bivariate analyses. For the consistency analysis, the *kappa* coefficient with quadratic weighting was used, interpreting the agreement findings according to the Landis and Koch scale (poor, 0–0.2; slight, 0.2–0.4; moderate, 0.4–0.6; substantial or high, 0.6–0.8; almost perfect, 0.8–1). A p-value of <0.05 was established as the level of statistical significance.

## Results

A total of 220 patients who presented any fracture were included. In total, 201 (91.4%) of patients presented a single fracture. The number of nonvertebral fractures was 206 (93.6%), and the number of vertebral fractures was 16 (7.3%). The mean age was 75.3±10.3 years (range: 60–103 years), and 150 (68.2%) were female. The inpatient mortality was 2.3% (n = 5).

A total of 55 (25%) patients had no prescription prior to hospital admission. The average number of drugs per patient increased from 3.2 (range 0–16) prior to hospitalization to 5.9 (range 1–16) at discharge. The highest proportion of patients with polypharmacy was identified during hospitalization (n = 217; 53.2%), while excessive polypharmacy was observed in 29 (13.5%) of the patient discharge prescriptions. The number of cases with polypharmacy increased from 38 to 92 cases (142.1%) at the time of discharge.

The anticholinergic burden scale that identified the highest number of drugs with antimuscarinic properties in the patients prior to being hospitalized was the ACB (26.8%), with quetiapine (6.8%) being the most prescribed drug. During hospitalization and at discharge, the ADS scale identified the highest number (77.7% and 72.1%, respectively), with a predominance of tramadol (64.1% and 61.4%, respectively). Using the ADS scale, an increase in the number of patients with an anticholinergic burden ≥1 at discharge was observed from 52 to 155 cases (198.1%) compared to that obtained in the month prior to the fracture. The increase was only 2 cases (3.3%) using the ACB scale and 2 cases (7.4%) using the ARS (Tables 1 and 2).

**Table 1:** Principal drugs with antimuscarinic properties identified with anticholinergic burden scales in patients with fractures treated in three hospitals, Colombia, 2018.

	ADS	n	%	ACB	n	%	ARS	n	%
**Before hospitalization (n = 220)**	Furosemide (L)	11	5.0	Quetiapine (H)	15	6.8	Quetiapine (L)	15	6.8
	Sertraline (L)	9	4.1	Metoprolol (L)	14	6.3	Carbidopa Levodopa (L)	6	2.7
	Nifedipine (L)	6	2.7	Furosemide (L)	11	5.0	Trazodone (L)	5	2.3
	Valproic acid (L)	4	1.8	Nifedipine (L)	6	2.7	Amitriptyline (A)	3	1.3
**During hospitalization (n = 220)**	Tramadol (L)	141	64.1	Ranitidine (L)	31	14.1	Ranitidine (L)	31	14.1
	Ranitidine (M)	31	14.1	Furosemide (L)	14	6.4	Quetiapine (L)	11	5.0
	Furosemide (L)	14	6.4	Metoprolol (L)	13	5.9	Metoclopramide (L)	9	4.1
	Sertraline (L)	9	4.1	Quetiapine (A)	11	5.0	Trazodone (L)	9	4.1
After hospitalization (n = 215*)	Tramadol (L)	132	61.4	Metoprolol (L)	17	7.9	Quetiapine (L)	13	6.0
	Furosemide (L)	13	6.0	Furosemide (L)	13	6.0	Carbidopa Levodopa (L)	6	2.8
	Sertraline (L)	12	5.6	Quetiapine (H)	13	6.0	Trazodone (L)	6	2.8
	Carbamazepine (M)	5	2.3	Trazodone (L)	6	2.8	Amitriptyline (H)	3	1.4

*5 cases of in-hospital death. ADS: Anticholinergic drug scales, ACB: Anticholinergic cognitive burden, ARS: Anticholinergic risk score, L: Low power. M: Moderate power. H: High anticolinergic power

**Table 2:** Polypharmacy and anticholinergic burden in patients with fractures treated in three hospitals, Colombia, 2018.

Variable	Before		During		After	
	n = 220	%	n = 220	%	n = 215*	%
Polypharmacy (5–9 drugs)	38	17.3	117	53.2	92	42.8
Excessive polypharmacy (≥10 drugs)	16	7.3	21	9.5	29	13.5
ADS ≥1 points	52	23.6	171	77.7	155	72.1
ADS 1–2 points	40	18.2	131	59.5	140	65.1
ADS ≥3 points	12	5.5	40	18.2	15	7.0
ACB ≥ 1 points	59	26.8	83	37.7	61	28.4
ACB 1–2 points	33	15.0	63	28.6	41	19.1
ACB ≥3 points	26	11.8	20	9.1	20	9.3
ARS ≥1 points	27	12.3	57	25.9	29	13.5
ARS 1–2 points	21	9.5	52	23.6	25	11.6
ARS ≥3 points	6	2.7	5	2.3	4	1.9

*5 cases of in-hospital death. ADS: Anticholinergic drug scales, ACB: Anticholinergic cognitive burden, ARS: Anticholinergic risk score

### Multivariate analysis

Through logistic regression, it was identified that presenting ≥5 drugs during the month prior to the fracture increased the probability of the patient having an anticholinergic burden of ≥1 point (ADS, ACB and ARS scale), while having 1–3 comorbidities and 4 or more comorbidities increased this probability according to the ADS and ACB scales, like being 85 years or older (ARS scale) (Table 3). Similarly, having 5 or more drugs at discharge increased the risk of having an anticholinergic load ≥1 point (according to the scales ACB y ARS) and have 4 or more comorbidities (scale ADS). On the other hand, according to the ACB and ARS scale, being a woman was associated with a lower probability of receiving an antimuscarinic drug. (Table 4).

**Table 3:** Multivariate analysis of the variables associated with the anticholinergic burden of prefracture prescriptions in patients treated in three hospitals, Colombia, 2018.

Variable	Sig.	OR	95% CI	
			Lower	Higher
**ADS ≥ 1 points**				
Woman	0.167	0.568	0.254	1.268
Age 60–74 years	0.710	Reference	Reference	Reference
Age 75–74 years	0.425	0.688	0.275	1.724
Age ≥85 years	0.600	0.785	0.318	1.938
Cartagena	0.246	1.829	0.659	5.081
0 comorbidities	0.002	Reference	Reference	Reference
1–3 comorbidities	0.001	6.503	2.073	20.397
≥ 4 comorbidities	0.001	12.673	2.902	55.343
≥ 5 drugs	0.001	4.638	1.862	11.554
**ACB ≥ 1 points**				
Woman	0.309	0.658	0.293	1.474
Age 60–74 years	0.689	Reference	Reference	Reference
Age 75–74 years	0.756	1.154	0.467	2.848
Age ≥85 years	0.388	1.482	0.606	3.622
Cartagena	0.876	1.083	0.399	2.939
0 comorbidities	0.058	Reference	Reference	Reference
1–3 comorbidities	0.027	3.276	1.147	9.354
≥ 4 comorbidities	0.033	4.542	1.126	18.325
≥ 5 drugs	<0.001	7.742	3.065	19.557
**ARS ≥ 1 points**				
Woman	0.424	0.651	0.227	1.866
Age 60–74 years	0.021	Reference	Reference	Reference
Age 75–74 years	0.480	1.571	0.449	5.500
Age ≥85 years	0.008	5.385	1.558	18.613
Cartagena	0.202	0.428	0.116	1.575
0 comorbidities	0.276	Reference	Reference	Reference
1–3 comorbidities	0.219	4.179	0.428	40.827
≥ 4 comorbidities	0.113	7.393	0.624	87.582
≥ 5 drugs	0.007	5.120	1.564	16.764

ADS: Anticholinergic drug scales, ACB: Anticholinergic cognitive burden, ARS: Anticholinergic risk score. Sig: Statistical Significance. OR: Odss Ratio 95%CI: 95% Confidence interval

**Table 4:** Multivariate analysis of the variables associated with the anticholinergic burden of outpatient prescriptions in patients with fractures treated in three hospitals, Colombia, 2018.

Variable	Sig.	OR	95% CI	
			Lower	Higher
**ADS ≥ 1 points**				
Woman	0.569	1.218	0.618	2.399
Age 60–74 years	0.227	Reference	Reference	Reference
Age 75–74 years	0.758	1.139	0.499	2.599
Age ≥85 years	0.145	0.566	0.264	1.216
Cartagena	<0.001	9.066	2.980	27.578
0 comorbidities	0.056	Reference	Reference	Reference
1–3 comorbidities	0.115	2.386	0.809	7.037
≥ 4 comorbidities	0.016	5.912	1.386	25.218
≥ 5 drugs	0.479	1.426	0.534	3.806
**ACB ≥ 1 points**				
Woman	0.041	0.413	0.177	0.962
Age 60–74 years	0.909	Reference	Reference	Reference
Age 75–74 years	0.719	1.172	0.494	2.782
Age ≥85 years	0.946	0.970	0.403	2.334
Cartagena	0.721	1.190	0.459	3.087
0 comorbidities	0.090	Reference	Reference	Reference
1–3 comorbidities	0.923	1.057	0.347	3.221
≥ 4 comorbidities	0.101	3.024	0.807	11.324
≥ 5 drugs	<0.001	22.162	5.240	93.73
**ARS ≥ 1 points**				
Woman	0.047	0.363	0.134	0.986
Age 60–74 years	0.179	Reference	Reference	Reference
Age 75–74 years	0.613	1.343	0.428	4.216
Age ≥85 years	0.072	2.775	0.913	8.434
Cartagena	0.140	0.392	0.113	1.362
0 comorbidities	0.250	Reference	Reference	Reference
1–3 comorbidities	0.607	0.641	0.118	3.491
≥ 4 comorbidities	0.508	1.815	0.311	10.585
≥ 5 drugs	0.019	16.040	1.579	162.973

ADS: Anticholinergic drug scales, ACB: Anticholinergic cognitive burden, ARS: Anticholinergic risk score. Sig: Statistical Significance. OR: Odss Ratio 95%CI: 95% Confidence interval

#### Consistency analysis

The degree of agreement between the scales evaluated varied with the medications used before, during and after the inpatient care. In prescriptions during the month prior to fracture, the lowest weighted *kappa* coefficient was found between the ADS and ARS scales (0.4879), and the best consistency occurred between the ACB and ADS scales (0.7171). Regarding the drugs administered during hospitalization, the lowest weighted *kappa* coefficient was found between the ADS and ARS scales (0.2584), and the highest consistency occurred between the ACB and ARS scales (0.6113). The consistency found with the drugs administered at discharge showed the best correlation between the ACB and ARS scales (0.5685) (Table 5).

**Table 5:** Comparison of the weighted *kappa* coefficient between 3 anticholinergic burden scales in patients with fractures in three hospitals, Colombia, 2018.

Comparison between scales	Before hospitalization			During hospitalization			After hospitalization		
	*Kappa coefficient*	*95% CI*		*Kappa coefficient*	*95% CI*		*Kappa coefficient*	*95% CI*	
		*Lower*	*Higher*		*Lower*	*Higher*		*Lower*	*Higher*
*ACB-ADS*	0.7171	0.6245	0.8097	0.3317	0.2471	0.4163	0.2766	0.1575	0.3956
*ACB-ARS*	0.5811	0.4671	0.6951	0.6113	0.5061	0.7165	0.5685	0.4447	0.6924
*ADS-ARS*	0.4879	0.3036	0.6722	0.2584	0.1781	0.3387	0.1274	0.0210	0.2337

ADS: Anticholinergic drug scales, ACB: Anticholinergic cognitive burden, ARS: Anticholinergic risk score. 95% CI: 95% Confidence interval

## Discussion

This study allowed documentation of the anticholinergic burden for patients during the month prior to a fracture, during hospitalization and at discharge, using the three most commonly validated scales and compared the degree of agreement between them, as well as the frequency of polypharmacy in this clinical context. These findings can be useful for scientific, academic and healthcare personnel because the results demonstrate the ability of each tool to identify the different antimuscarinic drugs, as well as the possibility of being interchangeable in patients with fractures, in order to make decisions clinics in a more homogeneous way. Using anticholinergic loading scales can help improve prescription quality by reducing polypharmacy, adverse reactions and harmful interactions in the elderly.

In the identification of anticholinergic prescriptions prior to fracture, the greatest consistency was found between the ADS and ACB scales (high: 71%), which was also evident in three other studies but in different clinical contexts (62% and 65% in patients with general illness in the US and Australia, respectively, and 62% in patients with dementia in Spain) [19, 20, 22]. The lowest consistency was observed between the ADS and ARS scales (moderate: 48%), differing from that reported in the other studies, where the poorest agreement was observed between the ACB and ARS scales (20%, 24% and 43%) [19, 20, 22]. In those studies, the consistency between ADS and ARS was 26% [19], 31% [22] and 53% [20].

The drugs with anticholinergic properties used during hospitalization gave levels of agreement that were different from those obtained in the prescriptions during the month prior to the fracture. The greatest consistency was evident between the ACB and ARS scales (high: 61%), followed by the ADS and ACB scales (slight: 33%) and finally between the ADS and ARS scales (slight: 25%), which is in contrast to the findings in a study conducted in patients hospitalized in a psychiatric institution in Spain, where significantly lower *kappa* coefficients (25%, 21% and 19%, respectively) were reported [21].

The discrepancies found in the consistency of the anticholinergic burden scales are due to various factors, among them, the different methodologies used in its elaboration and validation, the number of drugs that each tool includes, the presence or not of pharmaceutical forms that are used by ophthalmic or inhaled route, the availability or not of the drugs that include the scales in the market of the different countries, or the variations that exist in the degree of antimuscarinic potency of the drugs included in one or another scale [13–15].

In addition, the consistency of these instruments is due to the variation in the frequency of drugs used by patients in each of the various clinical contexts in which they are found. That is, drugs such as opioid analgesics that are very frequently used in the management of moderate/severe pain were only used in 3.6% of patients before presenting the fracture, but their use increased to 71.4% and 63.3%, respectively, in inpatient prescriptions and at discharge. Among those drugs, partial opioid agonists (tramadol, codeine) and total agonists (morphine, meperidine, fentanyl) have an anticholinergic burden according to the ADS [13] and ACB scales [15].

However, only the ADS scale includes tramadol in its list of anticholinergic drugs [13], marking a great difference with the other instruments, even more so considering that their prescription was found for 64.1% of hospitalized patients and 61.4% at the time of discharge. The use of opioids is related to adverse reactions such as confusion, hallucinations, delirium or sedation and therefore may be associated with an increased risk of falls [24]. In Colombia, it was found that the use of opioids (OR: 4.49, 95% CI: 2.72–7.42) was significantly associated with an increased risk of suffering a fall and presenting a hip fracture [25], which could happen with the opioids prescribed to hospital discharge, generating greater morbidity and mortality.

Because there are no studies that have quantified the agreement of the scales with the drugs prescribed at hospital discharge, a comparison was made with the drugs prescribed during the month prior to the fracture. It was found that the consistency of the three scales was lower for the outpatient prescriptions, with agreement between the ADS and ARS decreasing in 73.8%, between the ACB and ADS in 61.4% and with a minimum change of 2.1% between the ACB and ARS. These findings confirm that the presence of drugs that are only detected by one instrument and detected with great frequency, as in the case of tramadol, will affect the interchangeability of the scales, possibly in each of the different clinical contexts in which they are used.

Some limitations are recognized in the interpretation of certain results. The limitations are the small simple size and patients from only three hospitals were recruited, and the pattern and frequency of the drug prescriptions can change considerably between cities within the same country; therefore, it is necessary to perform a multicenter study to confirm the findings found in this report. There is also a chance that not all of the medications that the patient received prior to the fracture were identified. However, this study does compile a sample of patients from different levels of the Colombian health care system.

## Conclusions

From the above findings, we can conclude that in the clinical context of patients with fractures, these scales were not interchangeable, and the results were ostensibly modified at the different times when the prescriptions were evaluated, with the ADS scale being the tool that best identified antimuscarinic drugs. This result contrasts with the limited performance of the ARS scale.
